# Activating mutation in *MET *oncogene in familial colorectal cancer

**DOI:** 10.1186/1471-2407-11-424

**Published:** 2011-10-04

**Authors:** Deborah W Neklason, Michelle W Done, Nykole R Sargent, Ann G Schwartz, Hoda Anton-Culver, Constance A Griffin, Dennis J Ahnen, Joellen M Schildkraut, Gail E Tomlinson, Louise C Strong, Alexander R Miller, Jill E Stopfer, Randall W Burt

**Affiliations:** 1 Huntsman Cancer Institute, University of Utah, Salt Lake City, Utah, USA; 2 Department of Oncological Sciences, University of Utah, Salt Lake City, Utah, USA; 3 Department of Medicine, University of Utah, Salt Lake City, Utah, USA; 4 Karmanos Cancer Institute, Department of Medicine, Wayne State University, Detroit, Michigan, USA; 5 Department of Epidemiology, University of California Irvine, Irvine, California, USA; 6 Departments of Pathology and Oncology, Johns Hopkins University, Baltimore, Maryland, USA; 7 Department of Medicine, University of Colorado Denver, Aurora, Colorado, USA; 8 Department of Community and Family Medicine, Duke University, Durham, North Carolina, USA; 9 Department of Pediatrics, University of Texas Health Science Center at San Antonio, San Antonio, Texas, USA; 10 Department of Molecular Genetics, University of Texas M.D. Anderson Medical Center, Houston, Texas, USA; 11 Department of Surgical Oncology, START Center for Cancer Care, San Antonio, Texas, USA; 12 Abramson Cancer Center, University of Pennsylvania, Philadelphia, Pennsylvania, USA

## Abstract

**Background:**

In developed countries, the lifetime risk of developing colorectal cancer (CRC) is 5%, and it is the second leading cause of death from cancer. The presence of family history is a well established risk factor with 25-35% of CRCs attributable to inherited and/or familial factors. The highly penetrant inherited colon cancer syndromes account for approximately 5%, leaving greater than 20% without clear genetic definition. Familial colorectal cancer has been linked to chromosome 7q31 by multiple affected relative pair studies. The *MET *proto-oncogene which resides in this chromosomal region is considered a candidate for genetic susceptibility.

**Methods:**

*MET *exons were amplified by PCR from germline DNA of 148 affected sibling pairs with colorectal cancer. Amplicons with altered sequence were detected with high-resolution melt-curve analysis using a LightScanner (Idaho Technologies). Samples demonstrating alternative melt curves were sequenced. A TaqMan assay for the specific c.2975C **>**T change was used to confirm this mutation in a cohort of 299 colorectal cancer cases and to look for allelic amplification in tumors.

**Results:**

Here we report a germline non-synonymous change in the *MET *proto-oncogene at amino acid position T992I (also reported as *MET *p.T1010I) in 5.2% of a cohort of sibling pairs affected with CRC. This genetic variant was then confirmed in a second cohort of individuals diagnosed with CRC and having a first degree relative with CRC at prevalence of 4.1%. This mutation has been reported in cancer cells of multiple origins, including 2.5% of colon cancers, and in <1% in the general population. The threonine at amino acid position 992 lies in the tyrosine kinase domain of MET and a change to isoleucine at this position has been shown to promote metastatic behavior in cell-based models. The average age of CRC diagnosis in patients in this study is 63 years in mutation carriers, which is 8 years earlier than the general population average for CRC.

**Conclusions:**

Although the *MET *p.T992I genetic mutation is commonly found in somatic colorectal cancer tissues, this is the first report also implicating this *MET *genetic mutation as a germline inherited risk factor for familial colorectal cancer. Future studies on the cancer risks associated with this mutation and the prevalence in different at-risk populations will be an important extension of this work to define the clinical significance.

## Background

Colorectal cancer (CRC) is one of the more familial of cancers, and the presence of a family history of this malignancy is a well established risk factor. Twin studies suggest inherited and/or familial factors contribute to 25-35% of CRC cases [1]. The highly penetrant inherited colon cancer conditions including familial adenomatous polyposis, Lynch syndrome (hereditary nonpolyposis colorectal cancer), Peutz-Jeghers, Cowden syndrome and juvenile polyposis account for approximately 5% of CRCs, leaving greater than 20% without clear genetic definition [2]. An individual's risk of CRC doubles with one affected first degree relative and progressively increases with each additional affected first, second, or third degree relative [3 ,4].

**Figure 1:**
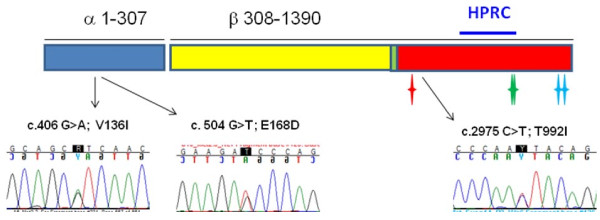
**Location of mutations identified in *MET *oncogene**. The MET protein is 1390 amino acids long (NM_000245.2, NP_000236.2). The pre-protein is cleaved into alpha and beta chains that are joined by a disulfide bond to create the extracellular receptor. The extracellular region (blue and yellow) contains a Sema domain, followed by a PSI and four IPT domains and are encoded in exons 2-12 of the mRNA. The transmembrane domain (green) is encoded in exon 13. The intracellular domain (red) is encoded in exons 14-21. Serine 985 (red star) down regulates kinase activity when phosphorylated, tyrosine 1234 and 1235 (green stars) positively modulate enzyme activity when phosphorylated, and tyrosine 1349 and 1356 (blue stars) recruit signal transducers when phosphorylated. Catalog of Somatic Mutations in Cancer (COSMIC) reports mutations in amino acids 130-370 of the extracellular domain, and 960-1340 of the intracellular tyrosine kinase domain. Amino acid changes responsible for Hereditary Papillary Renal Carcinoma (HPRC) are found between amino acids 1110 and 1268. Sequencing traces of the germline DNA mutations in the colon cancer sibling pair population are shown with their approximate location on the gene.

We have previously reported linkage of the 7q31 locus in a cohort of sibling pairs affected with CRC [5]. Other CRC and polyp-affected relative pair studies have also reported linkage to this region of chromosome 7 [6,7]. One candidate gene in this region is the proto-oncogene and tyrosine kinase receptor, *MET*. MET is expressed mainly on the surface of epithelial cells. In response to binding of the MET ligand, hepatocyte growth factor (HGF), C-terminal tyrosine residues are phosphorylated followed by a cascade of intracellular signals resulting in activation of MAPK and/or PI3K/Akt pathways [8,9] (Figure 1). In this way, aberrant activation of MET leads to increased cell proliferation, invasion, and metastasis [10,11]. The *MET *gene is found to be amplified in approximately 10% of CRCs, and amplification is associated with advanced stages and worse prognoses [12-14]. Additionally, *MET *gene missense mutations are found in ~3% of CRCs, in particular p.R970C and p.T992I (also reported as p.R988C and p.T1010I) [15]. Specific missense mutations in the tyrosine kinase domain of *MET *(amino acids 1112 to 1268) lead to hereditary papillary renal carcinoma (MIM ID 164860). Individuals with this condition are usually symptomatic by their fourth decade of life due to multifocal kidney lesions.

To evaluate the possibility that germline changes in the *MET *gene may lead to colon cancer susceptibility in a familial setting, we scanned and sequenced all coding exons of *MET *in our sibling pair cohort [5]. Here we report that *MET *p.T992I, a non-synonymous change suggested to have oncogenic potential, is present in the germline DNA of 5.2% of this cohort and this same change is confirmed in a second familial colon cancer cohort with two affected first degree relatives at a frequency of 4.1%. This substitution is reported in <1% of the general population and has been reported in cancer cells of multiple origins, including 2.5% of colon cancers [15-18].

## Methods

### Study population

All aspects of this study were approved by University of Utah's Institutional Review Board for human subject research. Research participants were consented to participate in a study of genetic factors leading to colon cancer risk. Ascertainment and collection of the sibling pair study cohort have been previously described [5]. Briefly, 169 siblings (148 affected with CRC and 21 unaffected) from families with two siblings diagnosed with colorectal adenocarcinoma or a polyp with high grade dysplasia and all of age > 20 years were included. A young onset population, defined as a CRC diagnosis ≤ 50 years (n = 130) and a familial colon cancer cohort, defined as two first-degree relatives diagnosed with CRC (n = 169), was used for confirmation of the *MET *p.T992I mutation. These populations have been previously described as well [19]. For both of these populations, diagnosis was confirmed with pathology report or state cancer registry data. Known syndromes were excluded through medical record review and/or molecular analysis including analysis for microsatellite instability (MSI) using the "reference marker panel" (BAT25, BAT26, D2S123, D5S346 and D17S250) and sequencing of normal tissue from cancers demonstrating MSI as previously described [5,19]. The presence of polyposis, defined as greater than 10 colonic polyps resulted in exclusion from the study.

### Scanning and Sequencing of *MET*

Primers were designed using Primer 3 [20] to PCR-amplify each of the coding exons of *MET*. Amplicons of 250-500 base-pairs in length were generated using LC Green dye mastermix (Idaho Technologies, Salt Lake City, UT, USA), for optimal high-resolution melt-curve analysis (HRM) using a LightScanner (Idaho Technologies). Samples demonstrating alternative melt curves were sequenced using Applied Biosystems 3730XL capillary sequencer.

### *MET *c.2975C > T specific TaqMan assay

Tumor DNA, micro-dissected and extracted from formalin-fixed paraffin-embedded (FFPE) blocks, was assayed for the presence of the *MET *c.2975C **>**T mutation (NM_000245.2, NP_000236.2), which results in p.T992I in the protein, using the Applied Biosystems probe assay for rs56391007 and TaqMan Genotyping Master Mix according to manufacturers' specifications (Applied Biosystems, Carlsbad, CA, USA). The major "C" allele was labeled with VIC (517 nm emission) and the minor "T" allele was labeled with FAM (554 nm emission). Products that peaked before 35 cycles using the BioRad CFX96 Real-time PCR machine with a signal of >500 RFU on the respective VIC or FAM channel were designated for that allele. When the *MET *c.2975C > T mutation was identified in tumors, normal DNA from the same patient (blood when available, otherwise FFPE micro-dissected normal) was tested for the mutation. Allelic imbalance was evaluated using cycle-time (CT) data generated by real-time quantitative PCR with the TaqMan assay. Where ΔCT = cycle time of VIC (c.2975C, wild type allele) - cycle time of FAM (c.2975T, mutant allele), with the fold excess mutant alleles = 2^(Δ CT tumor-Δ CT normal)^.

## Results

Because the *MET *gene resides within the linkage peak previously reported on chromosome 7q31 [5] and because it is an oncogene amplified in CRCs, the *MET *exons were scanned for variation using high-resolution melting (HRM) followed by sequencing of samples with different melt curves in our study population. The sibling pair population was composed of 169 subjects from 77 families including 148 affected with CRC and 21 unaffected [5]. The population is 86% Caucasian, 5% Black, 1% Native American, and 8% other. All known *MET *coding SNPs reported in dbSNP with population frequencies as well as coding variants identified in germline DNA of the sibling pair population but not present with population frequencies in dbSNP are listed in Table 1. The frequency of 9 of the SNPs in the sibling pair population are representative of the allelic frequencies reported in dbSNP and one unreported SNP results in a synonymous change (Table 1). Three non-synonymous *MET *gene changes were identified in the germline DNA of affected individuals whereby a population frequency was either not available or the minor allele frequency was less than 1% in dbSNP build 132 (http://www.ncbi.nlm.nih.gov/projects/SNP) [21]. Two individuals each had a single change in the extracellular domain of the MET receptor; p.V136I and p.E168D. However their affected siblings did not harbor the respective changes and the changes are not predicted to disrupt protein function with both Polyphen and SIFT analysis tools [22,23]. Seven of the 148 affected individuals, and none of the unaffected siblings, have the p.T992I germline change, which resides in the tyrosine kinase domain of MET. These 7 individuals represent 3 affected sibling pairs and 1 affected individual with an unaffected sibling or 4 of 77 families (5.2%). The consensus threonine amino acid at position 992 is completely invariant across vertebrates to zebrafish and both Polyphen and SIFT analyses predict that this change is damaging. This mutation has been previously reported in 2.5% of colon tumors [15]. It is reported in dbSNP at 0.7% based on low coverage sequence of ~600 individuals from the 1000 Genomes project (4 individuals). There are no clear pathologic characteristics of the individuals with p.T992I germline mutation (Table 2). The average age of CRC diagnosis in the sibling pairs with p.T992I germline mutation was 61.9 years with a range of 44 to 75 years, whereas the average age of the study population was 58.7 years (range 28-91).

**Table 1:** Allelic frequencies of germline variants in *MET *coding sequence for CRC sibling pair cohort and those reported in dbSNP

Exon	SNP*	Change*	# samples	CRC sibling frequencies	dbSNP allele frequencies
2	rs11762213	synonymous	160	89% GG, 11% GA	89% GG 11% GA

2	c.577C > T	synonymous	169	99% CC, 1% CT	Not reported

2	c.593G > A	V136I	169	99.4%GG 0.6%GT	Not reported

2	rs35775721	synonymous	165	88% CC 12%CT	97% CC 3% CT

2	rs55985569	E168D	165	99.4%GG 0.6%GT	99.5%GG 0.5% GT^#^

2	rs35776110	A320V	169	100% CC	97% CC 3% CT

2	rs77523018	M362T	169	98% TT 2% CT	98% TT 2% CT

2	rs33917957	N375S	169	100% AA	97% AA 3% AG

7	rs13223756	synonymous	161	75% AA 25% AG	67% AA 33%AG

14	rs56391007	T992I	163	95.7%CC 4.3%CT	99.3% CC 0.7%CT^#^

20	rs41736	synonymous	152	28% CC 54% GA 18%TT	37% CC 45% GA 18% TT

21	rs2023748	synonymous	157	26% GG 54% GA 20%AA	37% GG 45% GA 18% AA

21	rs41737	synonymous	157	26% GG 54% GA 20%AA	37% GG 45% GA 18% AA

*for variants without a SNP identification number in dbSNP, the nucleotide change is noted with reference to NM_000245.2 with nucleotide 1 referring to A of the AUG initiation codon and amino acid change is noted with reference to NP_000236.2. ^# ^Minor allele frequency from 1000 Genomes in dbSNP, no frequency data available under population diversity.

**Table 2:** Cancer cases with *MET *mutations

Sample*	Mutation	Age	Stage	Grade - differentiation	Colonic location	Relative copy number c.2975T (Tumor/Normal)
sib1	V136I	87	3	moderately well	transverse	ND

sib2	E168D	71	3	moderately well	splenic flexure	ND

sib3-A	T992I	52	2	well	rectosigmoid	1.60

sib4-A	T992I	62	1	moderately well	ascending	4.32

sib5	T992I	44	3	poorly	rectosigmoid	1.84

sib6-B	T992I	66	1	not reported	descending	1.79

sib7-B	T992I	66	1	not reported	descending	0.07

sib8-C	T992I	68	2	moderately well	cecum	0.31

sib9-C	T992I	75	1	moderately well	descending	0.38

hr10-D	T992I	71	4	moderate	cecum	0.06

hr11	T992I	60	1	moderate	splenic flexure	0.10

hr12	T992I	51	2	moderate	descending	0.34

hr13	T992I	77	2	well	sigmoid	0.05

hr14	T992I	89	2	moderate	rectum	2.55

hr15	T992I	35	2	moderate	transverse	1.56

hr16-D	T992I	47	4	moderate well	cecum	ND

hr17	T992I	68	3	moderate to focally poor	sigmoid	0.05

hr18	T992I	50	4	moderate	rectum	0.11

*sib indicates patient from sibling pair cohort, hr indicates patient from high risk cohort, -letter indicates same family. Not done (ND).

Because each of the families in the sibling pair cohort is of Caucasian descent, we were interested to determine whether there was evidence of a founder mutation. Using polymorphic markers surrounding the *MET *locus (D7S2418, D7S486 and D7S648), the haplotype segregating with the mutation in each of the 4 families was identified and found to be distinct. This suggests that this mutation arose independently.

Using tumor DNA from individuals diagnosed with CRC ≤50 years (n = 130) or individuals diagnosed with CRC and having a first degree relative with CRC (n = 169), a TaqMan assay was used to screen for the p.T992I mutation. We found that 9 of 299 tumors (3.0%) harbored the change, 1 from diagnosis ≤ 50 years (0.8%) and 8 from the two affected first-degree relative probands with CRC (4.7%). Interestingly, two of the 8 are a second-degree relative pair from a large family with excess colon cancer; one ascertained as a member of a first-degree relative pair (hr10-D on Table 2) and the other ascertained as a member of a parent-child pair also diagnosed ≤ 50 years (hr16-D on Table 2) [24]. Since these two are known to be closely related, the frequency is adjusted to 4.1% (7 of 168 families). DNA from normal tissue was available from all cases except hr16-D, and this individual was found to be an obligate carrier based on children's genotypes, thus the mutation was present in all 9 suggesting it was germline in origin. None of the 16 colorectal cancers arising in individuals with the germline p.T992I had microsatellite instability, a feature of defective mismatch repair pathway.

Because amplification of the *MET *gene occurs in colon cancers, we hypothesized that the allele harboring p.T992I would be preferentially amplified in colon tumors. Using cycle time from the TaqMan assay, we compared the difference of copy number of the mutant (c.2975T) from wild type (c.2975C) in tumor versus normal. Six of the 15 cases examined showed an excess of the mutant c.2975T allele over the wild type allele (Table 2), but this was not universal. This finding suggests that, in the presence of this mutation, additional amplification of the gene is not required for establishment of cancer.

## Discussion

Ten percent of all colon cancers arise in a familial setting when defined as two or more affected first-degree relatives [19]. There is a also two-fold increase of developing colon cancer with an affected first-degree relative [3]. Specifics of the genetic etiology of this group are not defined, leaving a gap in our knowledge of moderate risk genetic variants. A germline mutation in the *MET *gene, p.T992I, was identified in ~4.5% of colon cancers arising in first-degree relative pairs from two separate cohorts. The mutation was observed in less than 1% of colon cancer cases that occurred ≤ 50 years, suggesting that it does not promote very young CRC. *MET *p.T992I is also reported in the germline DNA of 4% of thyroid cancers [17], one endometrial and two melanoma cancer cases, and one normal individual [16]. In cancers, the p.R970C and p.T992I mutations are thought to affect phosphorylation of the serine residue (p.S985) that negatively regulates MET kinase activity [8,25]. By comparison, the specific missense mutations reported in hereditary papillary renal carcinoma (HPRC) surround two tyrosine residues within the catalytic site (p.Y1234 and p.Y1235) that positively regulate kinase activity (Figure 1). The phenotypic differences between p.T992I and HPRC *MET *mutations could be explained by the difference between factors driving negative versus positive activation of MET kinase.

We propose a model whereby the p.T992I mutation functions as a progression factor rather than an initiation factor in the canonical colon cancer model [26]. Specifically, we hypothesize that an adenoma is initiated through somatic mutations in the canonical APC pathway, then the adenoma acquires other proliferative mutations, and in the presence of an underlying *MET *p.T992I mutation, is then able to move beyond the mucosal layer to become invasive colon cancer. This hypothesis is supported by the following observations. The individuals we identified with the *MET *p.T992I germline mutation do not have the hallmarks of inherited mutations in initiating factors such as multiple adenoma formation (APC gene) or microsatellite instability (mismatch repair genes). Additionally, CRC diagnosis under age 50 is infrequent. In fact, the chromosome 7q genetic locus is associated in affected relative pair studies when CRCs are included and adenomas are excluded [5-7]. The average age of CRC diagnosis in the general population is ~71 years, and it is estimated that 10 years are needed for a small polyp to progress to invasive CRC [27]. A model of rapid progression of polyp to cancer in the presence of *MET *p.T992I is supported in that individuals with the p.T992I mutation are diagnosed with CRC at an average age of 63 years. This would be when the general population, on average, is developing adenomas that will progress to cancer. This mutation also occurs in a variety of cancers including colon, melanoma, endometrial, thyroid, and mesothelioma [15-18] with germline confirmation in colon, thyroid, uterine, and melanoma [16,17] suggesting that it is not a tissue-specific mechanism. *MET *p.T992I mutation is proposed to function through inhibition of phosphorylation of Ser985, which, when phosphorylated, corresponds with reduced MET signaling (Figure 1) [28,29]. In cell models, the specific mutation reported here generally affects invasive behaviors including changes in cell morphology, adhesion, motility, migration and anchorage-independent growth but not proliferation, such as IL-3 independent growth in Ba/F3 cells [18,29]. Based on these reports, it is reasonable to predict that MET p.T992I requires a growth signal (activation) but then is disabled in its ability to turn off the activation through phosphorylation of p.S985. It has also been shown that over expression of MET is an early event in the colorectal adenoma-carcinoma sequence [30]. In the context of a proliferating precancerous colonic adenoma, over expression MET p.T992I and the inability to turn off activation could allow the invasive behaviors to take place.

Although, the *MET *p.T992I genetic mutation has been commonly found in somatic colorectal cancer tissues, this is the first report also implicating this *MET *genetic mutation as a germline inherited risk factor for familial colorectal cancer. A strength of this study is the use of colorectal cancers enriched for a hereditary component. One of the limitations, however, is the small sample size and lack of a large unaffected cohort. Future independent studies on large case and carefully selected control sets are needed to replicate these results, confirm the conclusions, and provide an accurate estimate of the prevalence of this mutation in the cancer and normal populations.

## Conclusions

We estimate that the specific germline mutation of this investigation is responsible for ~4.5% of CRCs that occur in a familial setting, defined here as two first-degree relatives with CRC. Since 10% of all CRCs occur within this definition, this would translate to 0.45% of all CRCs. Future work should be focused on measuring the precise prevalence of this mutation in a large set of cases and controls and estimation of CRC risk associated with the germline mutation. Because *MET *p.T992I has also been found in germline DNA of individuals diagnosed with other cancers, a large-scale study to examine the penetrance and risks of all cancers in mutation carriers will be an important advance in our understanding. Specific inhibitors of the MET protein are currently in use in human clinical trials and may have a specific utility in preventing invasion and the metastasis of early-stage cancers in individuals with the *MET *p.T992I mutation [8].

## Competing interests

The authors declare that they have no competing interests.

## Authors' contributions

DN conceived of the study, its design, implementation, obtained funding and drafted the manuscript; MD designed and carried gene scanning and sequencing and assisted with manuscript; NS assisted with gene analysis, designed the TaqMan assays and analysis of tumors, and assisted with the manuscript; AS, HA, CG, DA, JS, GT, LS, AM, JS contributed samples and assisted with revision of the manuscript; RB participated in study design, obtained funding, and helped to draft the manuscript. All authors read and have approved the final manuscript.

## Pre-publication history

The pre-publication history for this paper can be accessed here:

http://www.biomedcentral.com/1471-2407/11/424/prepub
